# Development of a measurement system for complex oral information transfer in medical consultations

**DOI:** 10.1186/s12874-019-0788-7

**Published:** 2019-07-04

**Authors:** J. M. Nordfalk, P. Gulbrandsen, J. Gerwing, M. Nylenna, J. Menichetti

**Affiliations:** 1 Health Services Research Unit HØKH, Akershus University Hospital, Mail Drawer 1000, 1478 Lørenskog, Norway; 2 Institute of Health and Society, University of Oslo; Norwegian Institute of Public Health, PO Box 222, Skøyen, 0213 Oslo, Norway; 3 Institute of Clinical Medicine, University of Oslo, Akershus University Hospital, Mail Drawer 1000, 1478 Lørenskog, Norway

**Keywords:** Patient recall, Medical information, Measurement system, Physician-patient communication, Quantifying information, Information exchange, Shared decision-making, Multiple sclerosis, Escalation treatment, Unit of information

## Abstract

**Background:**

Information exchange between physician and patient is crucial to achieve patient involvement, shared decision making and treatment adherence. No reliable method exists for measuring how much information physicians provide in a complex, unscripted medical conversation, nor how much of this information patients recall. This study aims to fill this gap by developing a measurement system designed to compare complex orally provided information to patient recall.

**Methods:**

The development of the complex information transfer measurement system required nine methodological steps. Core activities were data collection, definition of information units and the first draft of a codebook, refinement through independent coding and consensus, and reliability testing. Videotapes of physician-patient consultations based on a standardized scenario and post-consultation interviews with patients constituted the data. The codebook was developed from verbatim transcriptions of the videotapes. Inter-rater reliability was calculated using a random selection of 10% of the statements in the transcriptions.

**Results:**

Thirtyfour transcriptions of visits and interviews were collected. We developed a set of rules for defining a single unit of information, defined detailed criteria for exclusion and inclusion of relevant units of information, and outlined systematic counting procedures. In the refinement phase, we established a system for comparing the information provided by the physician with what the patient recalled. While linguistic and conceptual issues arose during the process, coders still achieved good inter-rater reliability, with intra-class correlation for patient recall: 0.723, and for doctors: 0.761. A full codebook is available as an appendix.

**Conclusions:**

A measurement system specifically aimed at quantifying complex unscripted information exchange may be a useful addition to the tools for evaluating the results of health communication training and randomized controlled trials.

**Electronic supplementary material:**

The online version of this article (10.1186/s12874-019-0788-7) contains supplementary material, which is available to authorized users.

## Background

A key element of health care is for physicians to convey information about treatment choices in a way that patients can understand and later recall it. The importance of such information transfer can hardly be exaggerated, as it represents the condition sine qua non for success of care delivery [1]. Over the recent years, information exchange between physician and patient has become more complex, making methodological advances in understanding this issue not only relevant but also particularly challenging. Specifically, less paternalism, more transparency, and a higher degree of patient involvement in decisions are recommended [2–5].

Physicians today need to convey multiple, individualized information about uncertainty concerning prognosis, treatment effect, and risk of serious side effects, and at the same time take into account the need for more patient involvement in decision making, which leads to very complex information exchange. Health care providers need to adapt and keep up with the developing ethos.

Bridging the knowledge gap between physician and patient is challenging, even with less complex information [6]. In multiple studies, attempts have been made to evaluate patient recall [7–12]. Most of these studies considered less than 15 items of information given [8–10, 13–15]. Still, patients frequently forgot information within a short time span [8–11]. The amount the patients forgot was proportional to the amount presented [11, 14]. Patient recall has been shown to be less than 50% [10, 13].

It is reasonable to suspect that physicians frequently give patients a lot more than 15 items to remember. When too much complex information is presented, patients may become overwhelmed, rendering them less empowered to take part in the decision-making process [16].

As providing complex information is expected of physicians in medical encounters today, physicians require training on how to do it effectively within the demands of everyday practice. Training interventions require evaluation, generating the need to develop a method for measuring what patients have recalled. That is, to evaluate training interventions, we must be able to measure unscripted, complex information uptake reliably, comparing data from discussions during the encounter itself with data from the patients in a recall check.

In the literature, there are several types of tools or coding systems for measuring physician-patient communication. Most of these coding systems involve descriptive categorization. Among these, some split the interaction into different events to be counted [17], others look at who is talking [18], and what topics are being discussed [6, 19]. Linn and colleagues asked observers to mark on a checklist whether a topic and its subcategories were discussed during the consultation, comparing this list to patient answers on a recall questionnaire administered afterwards [12]. Finally, some look for specific phenomena with the aim of describing them [20].

There are also studies creating methods to assess the transfer of information quantitatively. However, most of these studies limited and/or strictly standardized the content of the information provided. Some of these studies departed from the arena of physician-patient consultation, instead imparting information to the patient from a list or an information movie, subsequently recording how much the patient remembered [11, 21–23]. A method made for strictly standardized contents may not be the best one to measure personally tailored complex information given in extemporaneous speech during dialogic interaction.

Furthermore, the definitions of “unit of information” in existing observational coding tools may limit the possibility of capturing complex information transfer. For example, with RIAS, Roter modified Bales’ process analysis scheme [24], by defining a unit of information as the smallest discriminable speech segment to which a rater can assign a classification and which expresses or implies a complete thought [25, 26]. Dunn and colleagues narrowed the definition further by defining a unit of information as “a segment of speech from the doctor expressing a single idea concerning medical issues” [27]. However, in complex information transfer there are sometimes speech segments that carry more than one idea, and have overlapping or mutually exclusive elements. There is information expressing insecurity, utterances like “if x, there is y % risk that z will happen”, and other types of rich, complex, borderline information-giving sentences. In addition, patients often paraphrase or simplify their recollections. To produce better solutions, we need to apply a complexity lens to our work [28].

Despite the plethora of observational tools for measuring physician-patient communication, there are no tools specifically developed to grasp the nuances of unscripted doctor-patient conversations during which they discuss complicated information. An exception is a recently published coding methodology aimed to measure patients’ memory of medical information delivered extemporaneously; this method, however, may not be widely applicable as it was developed on consultations requiring an interpreter. In addition, the authors themselves declared that the recall elicitation component may have been conducted too broadly and inconsistently [29]. Therefore, there is a need for improving existing measurement systems and providing new reliable methods specifically aimed at quantifying complex information giving as well as the patient recall rate in doctor-patient unscripted consultations.

This article reports the development of a complex oral information transfer measurement system involving the following: The definition of a unit of information, measurement of the number of such units regarding a chosen topic orally provided from physician to patient in a complex clinical consultation, and measurement of the number of units of this information that is recalled by the patient.

## Methods

**Fig. 1:**
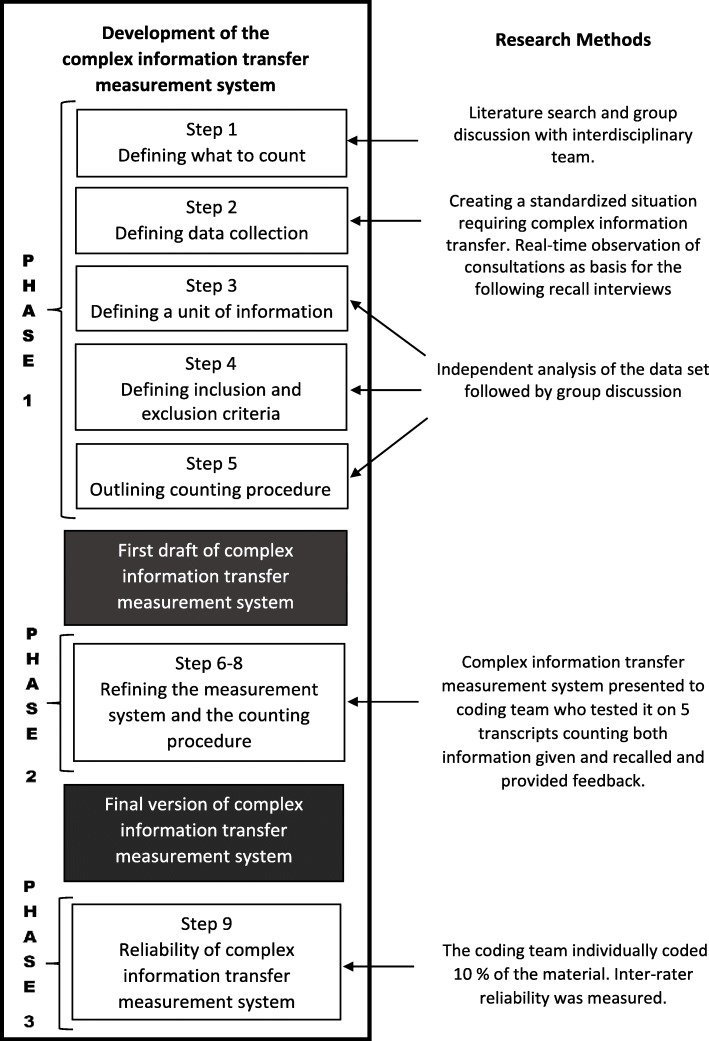
Methodological steps in the development of the complex information transfer measurement system

The development, refinement and reliability testing of the complex information transfer measurement system involved nine methodological steps: from defining the data needed for building this tool, to collecting it in form of video-recording standardized patient consultations as well as post-consultation interviews, and then to shaping the measurement system based on extensive analysis of the former. Figure 1 is an overview of the methodological steps.

*The first step* was identifying a clinical situation that would involve a complex exchange of information about medication. We decided to only count information focusing on the three most relevant drug alternatives when initiating second-line MS-treatment. To advise the patient in this choice entails conveying multiple, uncertain, situation-dependent – and thus complex - clinical information. Table 1 summarizes aspects of multiple sclerosis treatment that justify its choice as the clinical scenario for measuring complex information transfer.

**Table 1:** Aspects of initiating second-line Multiple Sclerosis treatment that makes the information exchange complex

Multiple Sclerosis-related aspects	
• Chronic disease	
• Unpredictable course	
• Potentially physically disabling disease	
• Affects cognitive functions	
Drug-related aspects	
• Available drugs differ in efficacy	
• Available drugs differ in risk/adverse effect profile	
• Available drugs differ in administration form	
• Long-term effects still unknown	
Individual-related aspects	
• Variability in the response to treatments	
• Variability in drug tolerance	
• Variability in health literacy	
• Variability in understanding of risk	

*The second step* was collecting data on the complex information exchange defined in step one. To achieve this, we needed to standardize the clinical setting, by creating a scenario in which initiation of second line treatment had to be discussed. Multiple sclerosis patients, currently on no or first line treatment, were instructed to imagine that they had had two recent attacks and had undergone an MRI-scan and blood tests. Therefore, they were now to consult with a neurologist to discuss the results and choice of further treatment. For all the other aspects, the patients were instructed to act as themselves. The same standardized case was given in advance to the neurologists, with specific clinical information and test results. They were also provided with an overview of information on the three most relevant second-line medications, natalizumab, alemtuzumab, and fingolimod, to compensate for differences in their level of experience. Each neurologist saw two patients, and all consultations were videotaped. All participants were recruited from the Neurological Department at Akershus University Hospital.

The interviewer (JN) observed the consultations on screen in real-time, using an observational sheet to register which information each physician conveyed to each patient. The observational sheet was developed to systematically keep track of the complex information conveyed, ensuring a tailored approach to the recall interview. Immediately following the consultation, JN conducted an individual recall interview with the patient. These interviews were also videotaped. The interviews were semi-structured and focused on drug information recall. The first part comprised open questions. Then, based on the notes collected during the observation of the specific consultation, JN narrowed the discussion to more detailed questions that were anchored specifically to the information the doctor had provided during the visit. All video recordings were transcribed verbatim. *The third step* aimed at describing how to identify and quantify unique units of information. We established a coding team of three members with experience from the fields of neurology, public health and communication. Outside the coding team, we had access to psychological and linguistic expertise. The first challenge was to define what a unit of information actually is. We pursued this through group discussion after first familiarizing ourselves individually with the transcripts. Subsequently, the three team members independently analyzed one randomly selected consultation. Then, group discussion revealed disagreements and areas of difficulty in the analysis and in the definition of “unit of information”. Discussion continued until they reached a consensus on the definition of unit of information. The same approach was used both in *step four* to reach agreement on inclusion and exclusion criteria for how to treat specific qualities of information (e.g., clarity, perceived medical importance, correctness of utterances) and in *step five* to outline the counting procedure. At that point, we were able to organize our decisions in a first draft of a manual for the reliable quantification of information units that were both conveyed by the doctor and recalled by the patient.

*In steps 6–8*, we selected five transcriptions, covering variations in the age and experience of the neurologists. Using the manual, all three coders independently counted all units of information delivered by the neurologists. They then analysed the corresponding five transcripts of the post-consultation interviews to count the patients’ recollections. Disagreements during the analysis process were resolved through group discussion, thus refining the analysis criteria and enabling the set of rules to cover as many of the problems that could arise as possible. This process was repeated four times, every time leading to revisions of the coding criteria and rules to make the analysis process as practically manageable and as reliable as possible.

*In the ninth step*, 10% of the information-carrying statements of each transcript was randomly selected and independently coded by the three coding team members, in order to calculate reliability.

## Results

The complete measurement system is shown in Additional file 1.

The following sections report the key results of the development process for the complex information transfer measurement system. These are organized following the methodological phases and steps described in the previous section.

### First phase: development (steps 1–6)

#### Data collection (step 1 and 2)

Out of 65 eligible MS-patients diagnosed in 2009–2012 at Akershus University Hospital, 42 agreed to participate. Thirty four finally participated; the others were excluded for practical reasons. Most of the patients were female (*n* = 25; 74%). The patients’ mean age was 46, median age 48 (range 29–66 years old).

Seventeen neurologists from the same hospital agreed to participate and to see two patients each. Most of the neurologists were male (*n* = 10; 59%). The neurologists had a mean age of 41, median age 39 (range 29–57 years old), and had between 2 and 29 years of work experience (median = 11, mean = 13).

All 34 consultations and interviews were transcribed. From the consultation transcripts, 1652 statements containing information about our predefined three drug alternatives were identified.

#### Defining a unit of information (step 3)

Initially, individual preferences regarding how much information to include in one countable unit of information differed considerably among the members of the coding team. To achieve concordance, the consensus was to count as a unit of information the smallest piece of information that still conveyed meaning. For example, in the statement «One option is Tysabri, which you get in a hospital as a monthly infusion. » the smallest possible units of information are:

- One option is Tysabri [a] *–name of medication 1p*
- In a hospital [b] *– administration place 1p*
- infusion [c] *– administration manner 1p*
- monthly [d]- *administration frequency 1p*

Therefore, four units of information are conveyed in this sentence, counting as 4 points for the “doctor’s information provision”.

#### Defining inclusion and exclusion criteria (step 4)

Following the development process, we defined a set of inclusion/exclusion criteria around overarching aspects:

- The doctor’s recommendation: We decided to include doctors’ opinions as they are a valuable piece of information for the patient to know (e.g. “If I were you, I would have gone for Lemtrada”).
- Incorrect information: Sometimes doctors conveyed medically incorrect information or information that was simplified to the point of being incorrect. We decided to include this type of information because the patient would not be able to discern between correct and incorrect information and would still need to process it.
- Importance of information: We decided to exclude the possibility of letting certain types of information be worth more points than others as defining “what is important and what is not” would have been not only a highly subjective task but would have implied a paternalistic approach.
- General information, in the sense of not specifically pertaining to one or more of the following three second-line multiple sclerosis-medications; natalizumab, alemtuzumab, and fingolimod, was excluded. We only counted information with sufficient contextual anchorage to be assigned to one or more of these specific drugs.
- Unclear, ambiguous, incomplete information: Information framed in a way that made it impossible to follow or interpret was excluded from being counted. Examples would be a sentence structure too fractioned to make sense, a double negation, or a lack of intrinsic meaning.

#### Outlining counting procedures (step 5)

**Fig. 2:**
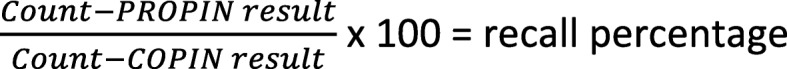
Calculation of recall percentage

We decided to start by counting the information units given by the doctor, and thereafter count the information units recalled by the patient, the latter to be considered a function of the first, see Fig. 2. This led to the development of a 2-step complex information transfer measurement system consisting of *“*Counting Complex Orally Provided Information” (Count-COPIN) and “Counting Patient Recall of Orally Provided Information” (Count-PROPIN).

### Second phase: refinement

In the three coders’ first attempt to apply the draft of the coding system to a subset of data, several aspects were found to require refinement and amelioration. These particularly focused on improving inclusion and exclusion criteria for information recall and optimizing the 2-step procedure of matching doctor’s information provision with patient’s information recall.

#### Improving count-COPIN in the complex information transfer measurement system

During the refinement phase, the coders decided not to count utterances with similar meaning twice, even when the doctor rephrased the information. In addition, if the doctor corrected her/himself, the coders decided to count only the last chronological piece of information. While repetitions stating a generalization or simplification were not counted additionally, if the repetition added new information or specified it, the coders agreed to count it additionally. The reasons for not counting repeats that do not add new information was that this would give the doctors a higher count, and thus unfairly reduce the patient recall rate.

Additional necessary precautions to avoid mistakes during counting are presented in the full manual (see Additional file 1).

#### Improving count-PROPIN in the complex information transfer measurement system

The application of the first version of the measurement system revealed specific situations to discuss (e.g., when the doctor listed specific points of information, but the patient remembered a general overview). It was decided to count all the mutually exclusive information units given by the doctor, and to give points to the patient for remembering generic overall information as well as specific details. For example, if the doctor gave a list of side effects, each item on the list earned a point. If the patient remembered all of them, each item on the list earned a corresponding point. However, if the patient only remembered that there were lots of side effects, this was awarded one point, as it is a unit of information remembered compared to not remembering anything about side effects. This raised the problem of how to treat, for example, a patient remembering that there were many side effects and then recalling some of the items listed. It was decided that a point would only be awarded for a recalled common denominator as long as not more than two individual items from a list were also remembered.

Furthermore, we decided that the patient would not be awarded points for producing information in the recall interview if:

- the information was not provided by the doctor during the consultation;
- the information was attributed to the wrong drug by the patient;
- the patient was clearly guessing.

An example of this last criterion was a situation in which the patient remembered a specific percentage, but she did not remember the particular context. The patient decided to give the same percentage as her answer to all questions concerning numbers, stating that this strategy would result in a correct answer to at least one question.

Finally, patients sometimes revealed prior knowledge of certain units of information. We decided not to remove points from the patient recall score for this. The reason behind this was the difficulty of verifying and discerning between previous knowledge and knowledge obtained during the consultation. .

#### Balancing the relation between count-COPIN and count-PROPIN

The material also offered situations in which the information was framed in an “if, then”-statement. Whereas for the physician, we decided to score only the parts of the whole, for patient recall, we decided to score both the parts and the whole (i.e., the relationship between the parts in an “if, then”-construction).

A final challenging aspect during the refinement phase was how to evaluate the patients’ understanding of the given information, differentiating between complete or partial understanding and evaluating whether the patient had achieved a good enough understanding. The most endorsed solution was: When in doubt, always err on the side of the patient.

E.g.: Physician: «Tysabri is given in hospital as a monthly infusion. »

[1p-name, 1p-location, 1p-frequency, 1p-admin. = 4p]

Patient recall when questioned on administration manner:

«It was in the blood once a month. »

[1p-frequency, 1p -admin. =2p]

In this case, the patient has already recalled the name of the drug in a previous utterance, so that information unit is already accounted for on the patient’s side. “Once a month” is an accurate recall of the doctors’ “monthly” =1p. The example is further meant to illustrate that we interpret “in the blood” as a good enough rephrasing of the information unit “infusion” =1p. We will count another point in the patient’s favour if she recalls that the drug needs to be administered in the hospital when answering the follow-up probing question.

### Third phase: establishing the inter-rater reliability

The intraclass correlation was excellent for COPIN; 0.761 and good for PROPIN; 0.723.

Table 2 shows relevant results when establishing inter-rater reliability.

**Table 2:** Interrater reliability of coders, based on 168 randomly selected statements comprising 10% of all statements in the material

	Coder A	Coder B	Coder C
Number of statements coded	168	168	168
Number of COPIN information units identified per statement (average, SD)	2.21 (1.43)	2.61 (1.95)	2.46 (2.05)
Number of PROPIN information units identified per statement (average, SD)	1.02 (1.13)	1.26 (1.34)	1.17 (1.28)
Overall ratio COPIN/PROPIN	0.46	0.48	0.47

The ratios of patient recall to information provided for the three coders agreed excellently. We used Bland-Altman plots to identify systematic bias. There was little such bias, which meant we could employ coders interchangeably.

## Discussion

This paper reports the development and reliability of a measurement system for complex medical information exchange. Unlike other coding systems that categorize contents [27, 30, 31], or describe interaction and count different types of talk occurring in the medical conversation [26], our measuring system counts the given and recalled units of information, without rating the quality, importance or correctness. This broadens the measurement system and gives it a potential to handle different kinds of complex information based on the topic under study. Even more, our measurement system overcomes important limitations in the literature as it offers a definition of unit of information that grasps the complexity of information exchange, thus improving methods for collecting patient information recall in unscripted conversations.

The main value of this measurement system is its ability to measure reliably both how many units of information on a pre-defined subject the physician has delivered to the patient, as well as how many of these given units the patient has in fact absorbed and recalled, thus providing a recall rate. It takes into account physicians’ repetitions and corrections and patients’ paraphrasing, generalizations and simplifications. It measures recall of the “gist” of the information, not only whether the patient is able to reproduce the doctor’s words exactly. Furthermore, the measurement system has been developed in a situation resembling real-life, particularly complex for what concerns the information exchanged. Therefore, it presumably fits real-life clinical conversations and the frequent situations during which the information is unsure, complex, individually adapted, and unscripted.

In previous studies on patients’ recall of information, recall is based on an often-limited amount of standardized information. Langewitz et al.’s study in 2015 [22] is an example of this, with 28 carefully chosen information units delivered. McCarthy et al. did two trials, delivering respectively 7 and 10 information units [15]. Sandberg did not test patient recall based on a personal medical conversation, but from an instructional video shown to all test subjects [21]. Our method differs from these studies as the amount of information was not limited a priori, nor was its content pre-determined. In a real-life medical consultations, the patient often receives a massive, complex and unselected amount of information, varying in clarity [32] and importance. The sheer amount of this information is likely to affect his or her recall [8, 9]. Hopefully, the information is also tailored to the patients’ specific needs, making it personally relevant. When patients expect an issue or a unit of information to have significant consequences for their own lives, they are more likely to become personally involved [33]. Consequently, the information is more deeply processed, and thus better recalled [34]. Our method contains a thorough definition of what a unit of information is, enabling quantification of any information deemed interesting to the research, embedded in complex free speech. This makes the measurement system well equipped for quantifying information in real-life conversations.

Another characteristic of our measurement system is the procedure to collect and evaluate patients’ information recall. The human mind can hold so much information, yet we access only a small part at a time. It has been demonstrated that contextual cues affect the ability to retrieve memory items and recall information in different situations [35]. Sandberg et al. compared recognition, free recall and cued recall; all methods used to measure recall in different studies [21]. Their study demonstrated that free recall is poor, but improves as more cues are provided. Performance on the multiple-choice task was better than cued recall performance, which was better than free recall performance [21]. In a recently published method for measuring information transfer, called PICcode [29], a short free recall interview was performed by research assistants who were not aware of the consultation contents. In our study, we wanted the preconditions to be as similar to a natural situation as possible. Therefore, our recall interviews were performed by an interviewer who witnessed the consultations in real time on-screen right ahead of the interview, and therefore was aware of which information had been given. This made it possible to achieve an intimately tailored interview with prompted recall, a technique placed somewhere in between free recall and cued recall. Since the interviewer had a checklist of which topics had been covered in the conversation, she was able to give open prompts, as a means towards making implicit knowledge explicit. With this procedure, the interviewer could ensure that the patients were prompted to search their memory about all topics mentioned by the doctor. Retrieval processes are cue-dependent: what we can and cannot recall at a given point in time is strongly influenced by the cues available to us [35]. If we had asked the patients to write down or just tell to the camera everything they remembered right after the consultation, it is probable that we would have gotten a much lower recall rate. If we had asked a fixed number of predetermined questions, we would not have achieved a reliable recall number for those doctors who had given more details or a higher number of information units. It is reasonable to assume that this tailored interview creates a more valid test of memory as it de facto works, by jogging the memory about each information unit given by the doctor, and that this would strengthen the reliability of the quantitative relationship between information given and recalled.

We believe that the ability of this measurement system to deal with complexity and provide a summative numerical output of complex information transfer makes it a useful tool for evaluating the impact of communication training interventions designed to improve complex information recall. The measurement system does not provide any kind of qualitative evaluation on the *manner* in which these units of information are delivered, it merely provides a numerical result. It could however be used in combination with other methods of categorization of doctor-patient interaction to see if recall percentage correlates with other communicational aspects. Having videotapes and transcripts available for linguistic analysis has the potential for furthering insight into how the details of communication increase recall rates. As an example, the measurement system could be adapted to investigate how increasing the use of repetitions as an information giving technique would affect patient recall.

The measurement system does not discriminate between information of different degrees of perceived importance, quality or correctness. It could be adapted to evaluating recall rate of all the above-mentioned types of information, but this would require a complementary development of a pre-defined information value scale that would vary with the individual, the chosen subject addressed in the consultation, and the prevailing medical paradigm in the actual practice. Moreover, it does not differentiate nor fully address the complex relationship between recall and understanding, even if it includes rules to credit recall when the information is heavily paraphrased, attempting to catch patient ‘gist’ understanding as well as more precise recollections. There is a recently published coding scheme that would be better equipped to detect mismatch between the intended meaning of the health care provider and the understanding of the patient [36].

This study has some limitations. First, choosing a standardized situation may have limited the generalizability of our findings to real-life situations.

However, the physicians reported that they found the situation realistic and recognizable. Furthermore, we recruited real MS patients, all in a stage where the fictitious situation was a realistic and foreseeable next stage of their disease. Nearly all patients confirmed that the information provided was relevant to them. Therefore, it is likely that the findings and the measurement system can be generalized and applied to real-life situations.

There is also a possibility of a Hawthorne effect; whether being observed has affected the behaviour of both neurologists and patients [37]. To minimize this possible effect, we used discreet ceiling-based camera equipment, and let the interviewer observe the consultation on-screen in an adjacent room. Neither physicians nor patients seemed to be affected by the cameras.

Another possible limitation of our study is that the reliability of the coding system was calculated on the results of three coders who were all involved in the development of the measurement system. Therefore, the coders were familiar with the problems and discussions preceding the decisions, which could have facilitated the reliability process and results. Further studies should strengthen the assessment of the coding system with external independent coders.

## Conclusion

We have developed a reliable method for measuring the information provided and recalled in a complex medical information exchange situation. It was designed for measuring recall in multiple sclerosis patients receiving information from a neurologist about their transition to second line treatment, but the method can potentially be adapted to other healthcare conversations involving complex information delivery. Furthermore, it can represent a reliable and useful tool for measuring the effect of communication training interventions on patient recall. We found high inter-rater reliability in this study. Further studies should follow to determine its reliability and validity in other clinical settings and care situations.

## Additional file

Additional file 1: Coding System for Counting Complex Orally Provided Information and Patient Recall (PDF 649 kb)

## Data Availability

The data owner is Akershus University Hospital. Requests for anonymized data should be directed to Professor Pål Gulbrandsen.

## Abbreviations

Count-COPIN: Counting Complex Orally Provided Information
Count-PROPIN: Counting Patient Recall of Orally Provided Information.
MRI: Magnetic resonance imaging
MS: Multiple Sclerosis
PICcode: Patient- Interpreter- Clinician coding
RIAS: Roter Interaction Analysis System
SD: Standard deviation
