# Supplementary material for: Development of a measurement system for complex oral information transfer in medical consultations
Source: BMC Med Res Methodol. 2019 Jul 4;19:139. doi: 10.1186/s12874-019-0788-7 (PMC6610985; doi:10.1186/s12874-019-0788-7)
Supplement: Supplementary file 1 — Coding System for Counting Complex Orally Provided Information and Patient Recall (PDF 649 kb) [file 12874_2019_788_MOESM1_ESM.pdf]

Coding System for
Counting Complex Orally Provided Information and Patient Recall

Count-COPIN: Counting Complex Orally Provided INformation

Count-PROPIN: Counting Patient Recall of Orally Provided INformation

By

Jenny M. Nordfalk[1], Pål Gulbrandsen[2], Jennifer Gerwing[3], Magne Nylenna[4]

[1] Jenny M. Nordfalk, Health Services Research Unit HØKH, Akershus University Hospital, Mail drawer 1000, 1478 Lørenskog, Norway, jenny.maria.nordfalk@ahus.no

[2]Pål Gulbrandsen, Health Services Research Unit HØKH, Akershus University Hospital, Mail drawer 1000, 1478 Lørenskog, Norway, pal.gulbrandsen@medisin.uio.no

[3]Jennifer Gerwing, Health Services Research Unit HØKH, Akershus University Hospital, Mail drawer 1000, 1478 Lørenskog, Norway, jennifer.gerwing@gmail.com

[4]Magne Nylenna, Institute of Health and Society, University of Oslo; Norwegian Institute of Public Health, PO Box 222 Skøyen 0213 Oslo, Norway, magne.nylenna@fhi.no

Introduction 3
General coding instructions using Count-COPIN and Count-PROPIN 4
2-STEP APPROACH 4
WHAT TO COUNT? 5
Count-COPIN: Counting Complex Orally Provided Information 6
1. INCLUSION OF INFORMATION 6
2. EXCLUSION OF INFORMATION 9
3. OTHER PROBLEMS 11
Decision Tree for analysis of the information provided by the doctor, sentence by sentence. 14
Count- PROPIN: Counting Patient Recall of Orally Provided INformation 15
1. Be liberal in favor of the patient when in doubt. 15
2. COUNT 0/1 15
3. COUNT n/n 16
4. The ideational content of a clause 18
5. Practical advice for using Count-PROPIN 18
Decision Tree for analysis of the information recalled by the patient. 19
References 20

Introduction

Physician engage in information transfer as a huge part of their communication with patients, both to learn facts about their patient, find out about their problems and symptoms, but also to educate, and impart knowledge and treatment advice.

Attempts have been made to study the effect of transferral of information, but no one has yet developed a reliable method of quantifying this phenomenon in unscripted consultations in which complex information transferral is necessary.

As providing information is a required and comprehensive part of medical encounters today, training physicians in how to do it efficiently is necessary. To evaluate training interventions, we need to be able to evaluate complex information uptake reliably, with data both from the encounter itself, and from patients thereafter. Hence, we needed to develop a method to do so.

This work aimed to describe the qualitative development of an information transfer measurement (ITM) coding system; a set of coding criteria for quantitative measurement of transfer of oral information from physician to patient in a complex clinical consultation.

The object of this manual is to describe a method of measuring the transfer of orally given information from physician (or other health care personnel) to patient; by a two step- method, first by identifying and counting all unique and meaningful units of information given by the physician during a consultation; then by identifying and counting the corresponding information units recalled by the patient in an immediately following interview.

Thus, we are able to find the proportion of information given that is actually absorbed and recalled by the patient.

General coding instructions using Count-COPIN and Count-PROPIN

2-STEP APPROACH

We advise a 2-step approach. It is imperative that the coder have read the entire manual before starting.

Step 1: The coder seeks to identify and count all unique and meaningful units of information presented by the physician, using the rule-set outlined in Count-COPIN (Counting Complex Orally Provided Information)

Step 2: The coder seeks to identify and count all unique and meaningful corresponding units of information recalled by the patient, using the rule-set outlined in Count-PROPIN (Counting Patient Recall of Orally Provided Information).

$$\frac{Count-PROPIN\ result}{Count-COPIN\ result} \text{ x 100 = recall percentage}$$

WHAT TO COUNT?

As a coder, you need to define what you want to measure, and what kind of data you will need to collect to achieve this. This will of course vary according to what kind of research you are partaking in. It is necessary to make overall decisions in advance on what to include and what to exclude, to keep the material from becoming too large and impossible to handle.

In the study we used to develop this method of measuring the transfer of orally given information, we chose the possible initiation of second line Multiple Sclerosis (MS) treatment as case due to its extreme information complexity and high inherent uncertainty. MS patients currently on no or first line treatment were instructed to imagine having had a history of new attacks, before fictively having had an MRI and blood samples taken. They patients then consulted with a neurologist to receive information about these fictive test results and discuss the choice of further treatment. Immediately after the consultation, a researcher conducted a patient recall interview. Consultations and interviews were videotaped and transcribed verbatim.

During the development of the coding criteria, we chose to only count information pertaining to the following three second-line Multiple Sclerosis-medications; Lemtrada, Tysabri and Gilenya. This is reflected in the inclusion criteria in chapter 2, 1) A and the exclusion criteria in chapter 2, 2). If you wish to focus on a different informational content, you need to adapt these criteria accordingly.

To ensure reliability between coders, follow the rules given in Count-COPIN and Count-PROPIN.

Count-COPIN: Counting Complex Orally Provided Information

Method of identifying all unique and meaningful information units about relevant medication presented by a physician in dialogue with a patient.

1. INCLUSION OF INFORMATION

1.1. We include the following information about the three drugs we wish to count information about:

1.1.1. Reasons for use

1.1.2. Effects

1.1.2.1. This includes working mechanisms.

1.1.3. Side effects

1.1.3.1. This includes counting information about blood testing for JCV-antibodies and the risk for progressive multifocal leukoencephalopathy (PML) a positive test implicates.

1.1.4. Prerequisites for use including up front testing, procedures etc.

1.1.5. Precautions

1.1.6. Administration

1.1.6.1. Infusion, injection, oral, suppository, others

1.1.6.2. Frequency

1.1.6.3. Dosage

1.1.6.4. Place

1.1.7. Recommendations

1.1.8. Comparisons

1.1.8.1. Equal information about two or more drugs provided in the same sentence.

E.g.: «Both drugs have clearly beneficial effects» (said about Gilenya and Lemtrada, which is clear from the context).

The information is:

→ Both drugs [a] 1p

→ have beneficial effects [b] 1p

= Count 2 points

1.1.8.2. Comparative information about two or more drugs provided.

E.g.: Interview 32 (I-32): «about Gilenya, it is also very effective, but Tysabri seems likely to have a somewhat better effect»

Count 2p; 1p for the information that Gilenya is effective, and 1p for Tysabri being seemingly more so than Gilenya. Do NOT in addition count 1p for Gilenya being less effective than Tysabri. However, be aware that the patient may recall the information in this manner.

1.1.9. General statements or characterizations of two or more of these drugs

1.1.9.1. Information about two or more medications as a group

E.g.: I-19: «All three treatments are well documented and efficient»
When the information is given as a group, we count it as a group; 1p for «all three», 1p for «well documented», 1p for «efficient».

1.2. We count all meaningful/useful information.

1.2.1. We have found that the only possible way to count the information reliably is to break it down in to as small units as possible while still maintaining useful information.

E.g.: «One option is Tysabri, which you get in a hospital as a monthly infusion. »

Here we are exemplifying how to break the statement into countable units of information:

→ One option is Tysabri [a] – *option 1p*
→ In a hospital [b] – *administration place 1p*
→ infusion [c] – *administration manner 1p*
→ monthly [d]- *administration frequency 1p*
= 4p

1.2.2. For a unit of information to be useful, it needs to contain a subject, a verb, and at least one of the following; object, complement, or adverbial. This limits the size of our units of information.

E.g.: I-19 «So it is sort of the three main treatments that are relevant»
Underneath we are exemplifying two different ways of breaking the statement into countable units of information:

Example 1.

→ three main treatments are relevant [a]-*general statement, no. of relevant main treatments* 1p

If we try to break it into smaller pieces, we could get something like this:

Example 2.

→ three [a]- 1p
→ main [b]- 1p
→ relevant [c] -1p

But all of them would be useless information on their own. That is why we give only one point, like in example 1.

1.2.3. Still, there can be more than one unit of information hiding in one clause. The ideational content of a clause can involve four types of constituent that: processes (actions/ events/ states), participants (persons/ things/ abstractions), qualities/ states/ features pertaining to the participants, and circumstances (time/ manner/ place/ reason, etc.) of the process and the participants.[1]

E.g.: I-27 «Gilenya is a pill, which you take daily. » Here we are exemplifying how to break the statement into countable units of information:

→ Gilenya [a] – option (participant) 1p
→ Is a pill [b] – administration manner (quality) 1p
→ Which you take daily [c]- administration frequency (event) 1p
= Count 3 units of information 3p

In all the examples above, a point has been given for the name of the medication as the «participant". A point for the information of the name «Gilenya", however, will only be given once throughout the entire transcript. It is necessary to avoid double scoring for repeated information. This will be addressed below in Count-COPIN 3.2.1.

1.3. If the information is incongruent within a statement or with a previous statement, both units of information are to be counted; unless the doctor obviously corrects herself, see example in Count-COPIN 3.4.1.

1.4. When both generalized and specialized information are presented, they are counted separately.

1.5. When the doctor confirms, denies or corrects a statement or question from the patient, this is to be counted as information provided.

2. EXCLUSION OF INFORMATION

These exclusions are specifically tailored according to the study used for developing this manual. If you want to count different information, you need to adapt these criteria accordingly.

The transcriptions we worked with when developing this coding system stem from standardized consultations between neurologists and MS patients about initiating second-line treatment, with focus on the three most common options in Norway at the time. The patients were told to imagine that they had deteriorated on the treatment they were currently receiving.

2.1. We exclude non-medical information or information unrelated to the three drugs we have chosen to focus on.

2.1.1. All information that could not be specifically assigned to one or more of the three drugs we have chosen to focus on is to be excluded

E.g.: I-19 «there are a lot of new medications, which makes the choice more difficult, since we have a lot to choose from»

Deemed to be too general statements, and nor specifically referring to our three chosen medications.

E.g.: I-30 «So, in reality we have three options. » - 0p

No points for the doctor in this example, as the three options will be mentioned and scored for separately. The patient however will get points for remembering that there were three options, if he or she does not remember the separate options.

2.1.2. All information referring just to the term second-line treatment is to be excluded.

Reason: to make the body of information manageable within a reasonable time frame.

E.g.: I-24 «Generally, second-line treatment is more effective than first-line treatment, right? »

2.1.3. All information involving Tecfidera and stem-cell treatment is to be excluded.

Reason: The interviewer does not ask the patient about recollections of this information.

2.1.4. All information about failure of previous medication (first-line medication), as well as information about MRI or clinical progression is to be excluded.

Reason: The information is fictive, and the recall interviewer does not ask about recollections of this information.

E.g.: I-5 «from what we gather from the pictures, and from how you have been the last year, it seems like the treatment you have had, has been a little too inefficient for you, »

2.1.5. Information about having taken a blood sample is to be excluded.

Reason: This is something the patient already knows, and the interviewer does not ask about. The result of the sample is relevant information that is to be included in the counting, but not the statement that the sample has been taken/results are present.

E.g.: I-23 «Um, first I need to tell you that we have also received the result of that blood sample, which is important to consider here, that is, that you are positive to the JC-virus... this index. »

→ I need to tell you – too general, not medical information -0p
→ We have received a result of the blood sample – information already known and not to be counted 0p
→ Important to consider – side effects 1p
→ You are positive - side effects 1p
→ JC-virus – side effects 1p
→ Index – side effects 1p
= Count 4 units of information 4p

2.2. We exclude too general information.

2.2.1. Information that is too general to be deemed medical information is to be excluded.

E.g.: I-30 «It is important that you are familiar with the treatment» Deemed general conversation, not medical information, thus not to be counted.

2.3. We exclude information without sufficient contextual anchorage, e.g. utterances that cannot contextually be assigned to specific drugs

2.4. We exclude ambiguous information.

2.5. We exclude start-up information related to the design of our study.

1. Practical start-up information encompasses fictive plans to meet again at a certain interval, letters, brochures and planned follow-up phone calls or contact with other health-care personnel, etc.
Reason: Fictive future plans are not relevant for the patient to remember. The interviewer does not focus on this information during the recall interview.
E.g.: I-22 «, and that we will talk again in about two weeks. » -0p

2. Practical start-up information does not encompass specific examinations or tests that are needed in order to start with a new medication. This falls under Count-COPIN 1.1.4. «Prerequisites for use» or 1.1.5. «Precautions».

3. OTHER PROBLEMS

3.1. Equality of units of information

3.1.1. We do not rate or discriminate according to the perceived importance of the information.

3.1.2. We do not rate or discriminate according to the perceived correctness of the information.

3.1.3. We do not rate or discriminate according to the perceived quality of the information.

3.2. Repetitions / information with similar meaning: It is necessary to avoid double scoring for repeated information in the entire consultation. The coder needs to have this in mind and check for repetitions not only within a sentence, but also throughout the transcript.

3.2.1. If the repetition gives precisely the same information, do NOT count.

E.g.: I-27 (abridged) «The thing with Gilenya is that it may slow the heart rate. »

→ Gilenya [a] – option (participant) 1p
→ may slow the heart rate [b] – side effect (quality) 1p
= Count 2 units of information 2p

This affects the counting in the following sentence in the transcript:

E.g.: I-27 «Gilenya is a pill, which you take daily. »
Here we are exemplifying how to break the statement into countable units of information:

→ Gilenya [a] – option (participant) /<u>REPETITION</u>
→ Is a pill [b] – administration manner (quality) 1p
→ Which you take daily [c]- administration frequency (event) 1p
= Count <u>2</u> units of information <u>2p</u>

3.2.2. If the repetition gives precisely the same information, even if with other words, do NOT count.

E.g.: I-33 «Also, everyone gets a rash... heh, 98 % get a rash, that is, only while receiving the treatment»

→ Also, everyone -[a] 1p
→ gets a rash... [b] 1p
→ heh, 98 % [c] *correction - no extra point*
→ get a rash [d] *repetition – no extra point*
→ that is, only while receiving the treatment [e] 1p
= 3p

Then when we look at some other related statements in I-33: «that is associated with that treatment only... » and «but it is temporary»

→ that is associated with that treatment only... *Repetition of information [e] though in other words - Count no additional point.*

→ but it is temporary - *Repetition of information [e] though in other words - Count no additional point.*

3.2.3. If the repetition gives a somewhat added or altered information, count.

E.g.: I-33 «Also, everyone gets a rash... heh, 98 % get a rash, that is, only while receiving the treatment»

→ Also, everyone -[a] 1p

→ gets a rash... [b] 1p

→ heh, 98 % [c] *correction - no extra point*

→ get a rash [d] *repetition – no extra point*

→ that is, only while receiving the treatment [e] 1p

= 3p

In another sentence in I-33: «yeah, I think it is a so-called generalized rash, »

→ <u>generalized</u> rash [a] 1p *added information*

= 1p

3.2.4. If the repetition states the same in an easier or more general manner than previously provided, do NOT count.

E.g.: I-19: «And that is <u>close to 50%</u>, so <u>that is a good share</u> »

→ close to 50% [a] 1p

→ that is a good share [b] *repetition/simplification – no extra point*

= 1p

E.g.: I-19: «But <u>if you have,</u> for example, are very strongly positive and have used it for many years, <u>then it will</u> increase from 3 per 1000 up to 10 per 1000 every single year <u>so that would be many times higher»</u>

This is also a good example on how to break the statement into units of information:

→ If you are very strongly positive [a] 1p

→ and have used it for many years [b] 1p

→ then, it will increase [c] 1p

→ from 3 per 1000 [d] 1p

→ to 10 per 1000 [e] 1p

→ every single year [f] 1p

→ so that would be many times higher [h] 0p We see [h] as a r*epetition/simplification of the information in [d] and [e]– no extra point*

= 6p

3.3. «If, then» – expressions

3.3.1. Count the units of information, but do not in addition count the whole. (In Count-PROPIN on the other hand, the patient may be awarded points both for understanding the whole and for remembering loose units of information.) So, no extra point for the «if, then»-relationship between the two statements in Count-COPIN.

E.g.: I-19 «Worst case scenario, if your metabolism becomes chronically low, you will need to take pills to get up on a normal level. »

→ Worst case scenario -[a] 1p
→ If your metabolism becomes chronically low [b] 1p
→ You will need to take pills [c] 1p
→ to get up on a normal level [d] 1p
= 4p

3.4. Corrections

3.4.1. If the physician provides information, then later explicitly corrects her/himself, count ONLY the last version of the information.

E.g.: I-33: «Also, everyone gets a rash... heh, 98 % get a rash…»

→ rash [a] 1p,
→ everyone [b] 1p.
→ heh, 98 % The *physician corrects herself, see* [b]. *Count no additional point. The information of 98% stands.*
→ get a rash *Repetition of* [a]. *No additional point.*
= 2p

3.5. Information provided with words of low specificity.

3.5.1. Evaluate the distinction between the words and do not count excessively

E.g.: «You will feel weak, indisposed, knocked out". =1p

Decision Tree for analysis of the information provided by the doctor, sentence by sentence.

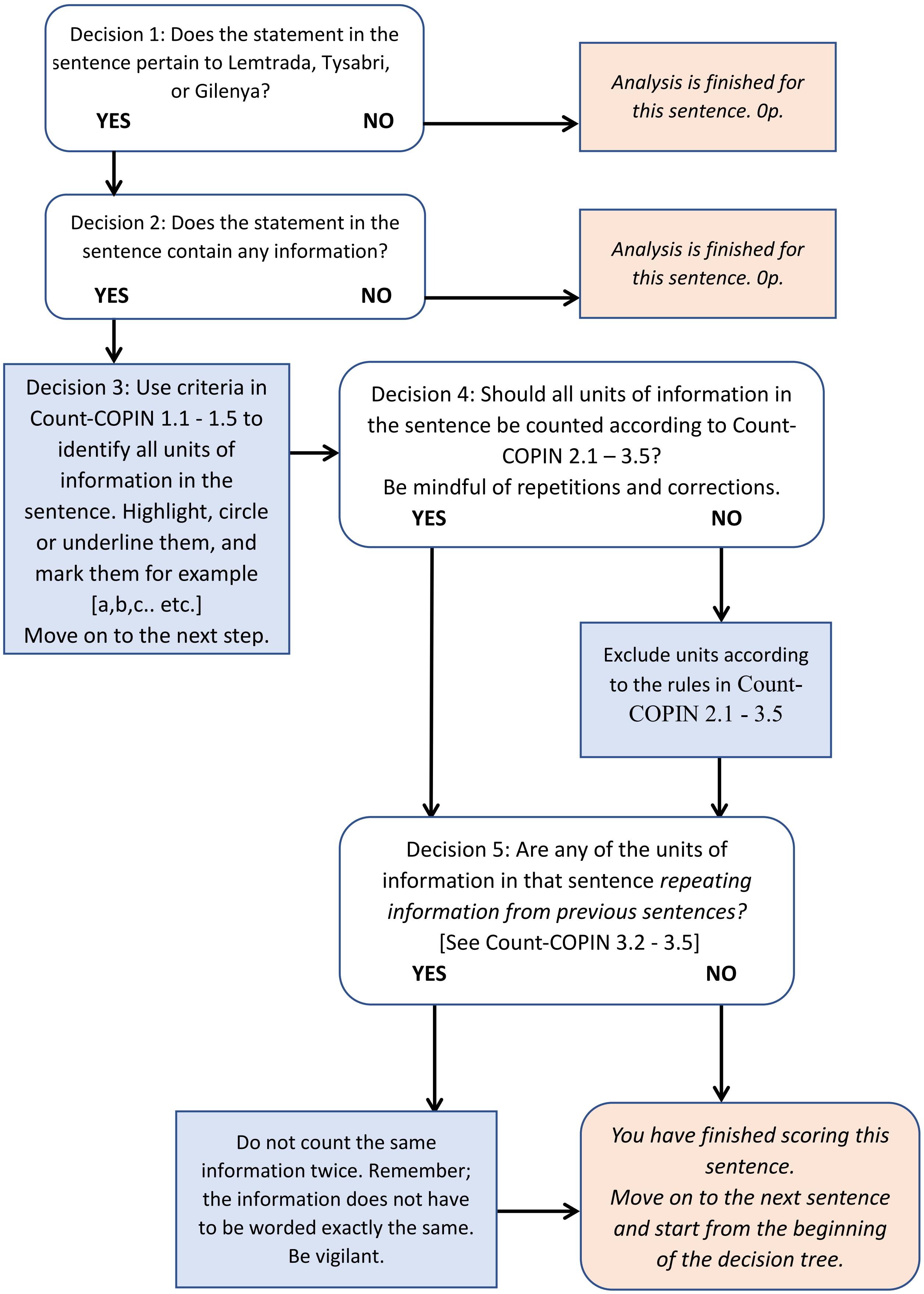

Count- PROPIN: Counting Patient Recall of Orally Provided INformation

Method of identifying all unique and meaningful corresponding units of information recalled by the patient, following orally provision of information about relevant medication by a physician.

To use Count-PROPIN we assume that the coder has already used Count-COPIN, thus having already defined the units of information provided and counted them.

1. Be liberal in favor of the patient when in doubt.

There is a theory that doctors in general give too much information. To ensure that we do not design the measurement tools to reinforce our own prejudices, we wish for the coders to be liberal in favor of the patient.
When the patient recalls, the words will often be somewhat rephrased, generalized and altered. If the coder's interpretation is that the patient has grasped the message and is able to rephrase it, we give points liberally.

E.g.: I-19 «So it is sort of the three main treatments that are relevant»

→ three main treatments are relevant[a] -*general statement, number of relevant main treatments*

-0p for the doctor in this example, because the three relevant options were mentioned and scored for separately. (See Count- PROPIN 3.2 below)
(1p would have been counted for the doctor mentioning that there are three options - if the three options had not been scored for separately.)

When the patient recalled this, in this example, she said: «There were three options here."

→ three options 1p.

2. **COUNT 0/1** (zero/one possible) if
   - 2.1. The patient attributes the information to the wrong drug.
     - 2.1.1. Exception: See «Avoid downstream errors" below in Count-PROPIN 3.3.
   - 2.2. The patient is not able to provide a clear response.
   - 2.3. The patient demonstrates lack of understanding of information
   - 2.4. The patient says s/he doesn't remember
   - 2.5. The patient is clearly guessing.

3. **COUNT n/n** (number of units/number of units possible)

3.1. Correct patient recall of any bit of information provided and counted using Count-COPIN

3.2. Generalized vs. detailed recall.

3.2.1. Situations: The doctor provides a list of side effects. The doctor informs about how many side effects there are, before giving detailed information about each option.

All items on a list that the patient recalls will count as 1p. each.

If the patient remembers a common denominator, e.g.: «there were plenty of side effects", this will count as 1 point.

If the patient remembers a common denominator AND individual items on the list, the recall of the common denominator will only earn a point as long as the patient does not remember more than two individual items on the list.

If the patient remembers three or more side effects AND says: «there were many side effects", score NO additional point for the latter.

E.g.: The doctor says «you will have to do monthly (1) blood (1) and urine (1) tests, where they check for blood platelets (1) and thyroid function (1), and also an MRI scan (1) biannually (1)" - total 7p.

→ Alt. 1) The patient recalls «there was a lot of follow-up" –1p

→ Alt. 2) The patient recalls «there was a lot of follow-up, like blood samples" –2p

→ Alt. 3) The patient recalls «there was monthly (1) blood (1) and urine (1) tests and an annual MRI scan (1)" – total 4p

→ Alt. 4) The patient recalls «there was a lot of follow-up, there was monthly (1) blood (1) and urine (1) tests and an annual MRI scan (1)" Give no additional points for the generalization, as the patient recalled more than two items from the list – total 4p

→ Alt.5) If the patient recalls all items on the list -total 7p.

→ Alt.6) If the patient recalls all items on the list + says that there was a lot of follow up-total 7p.

3.2.2. Avoid counting a unit of information more than once.

E.g.: If the patient recalls that there were three options, and then proceeds to mention all three options, (s)he should not get a point for the first answer. If (s)he only remembers two of the options, but remembers that there existed a third, this point is relevant to count.

3.2.3. The patient remembers a generalization from many things the physician has said.

E.g.: The physician explains in great detail about the patient carrying a low level of a virus antibody in their body, and that there is a risk that using Tysabri may reactivate this virus and lead to a serious brain infection. The level of these antibodies may increase, which would increase the risk of this dangerous side effect.

I-4: The patient recalls «there was something about some antibodies »

The patient should get 1 point for remembering that the antibodies were mentioned.

3.2.4. «If, then" – expressions: The patient may be awarded points both for understanding the whole and for remembering loose units of information.

E.g.: I-19 «Worst case scenario, if your metabolism becomes chronically low, you will need to take pills to get up on a normal level. »

→ Alt. 1) The patient recalls «worst case, you had to take pills» – 2p
→ Alt. 2) The patient recalls «It could affect your metabolism" –1p
→ Alt. 3) The patient recalls «worst case (1) low metabolism (1) treatable (1) with pills (1)" – total 4p
→ Alt. 4) The patient recalls «If things got really bad, you could end up having to take other pills for this» If-then (1) pills (1) – 2p

3.3. Avoid downstream errors.

3.3.1. We do not want real transfer of knowledge to be camouflaged as downstream errors. E.g.: A patient cannot initially remember the name of a drug, but s/he recalls how the different drugs were administered. Interviewer and patient agree to refer to the drug as «the second drug". The patient recalls multiple units of information about the drug. The patient should get the adequate points for this.

4. The ideational content of a clause

4.1. When the coder is working with the task of identifying the primarily given information in the transcript of the post-consultation interview with the patient, we advise the coder to look for the ideational content of a clause. This can involve four types of constituents: processes (actions/ events/ states), participants (persons/ things/ abstractions), qualities/ states/ features pertaining to the participants, and circumstances (time/ manner/ place/ reason, etc.) of the process and the participants.[1] These categorizations can be used as an additional tool by the coder as an aid to help single out the units of information.

E.g.: Count-COPIN [I-27-47] «Gilenya is a pill, which you take daily. » Here we are exemplifying how to break the statement into countable units of information:

→ Gilenya [a] – option (participant) 1p
→ Is a pill [b] – administration manner (quality) 1p
→ Which you take daily [c]- administration frequency (event) 1p

= Count 3 units of information 3p

5. Practical advice for using Count-PROPIN

The coder is advised to keep the consultation transcript with his or her notes present while going through the interview transcript. When the patient manages to recall any of the units of information given by the physician, this should be marked; e.g. with which line in the consultation transcript the unit of information was given in and marked with a),b) or c) to keep track of which unit of information in the sentence they stem from.

Decision Tree for analysis of the information recalled by the patient.

Decision 1: Look for presence of relevant information

Decide where the useful information in the transcript is (i.e., eliminate/cross out sections without information, and underline or mark information pertaining to the three medications we are studying.) Distinguish between information and non-information in some way that works for you.

→

Decision 2: Matching of information

- Follow the rules in Count-PROPIN.
- Refer both to consultation transcript and the interview transcript to catch anything that is <u>unique</u> information from the patient that matches unique information from the doctor.
- Traceability is important: Circle, underscore or highlight in the interview transcript and give that circled information the corresponding code as you gave the information in the consultation transcript.

↓

Decision 3: Are any of the matching units of information repetitions?

Be vigilant. [See Count-PROPIN 1 - 4]

↓

Decision 4: Make sure to go back and correct previously given points when necessary.

[See Count-PROPIN 3.2]

↓

Decision 5: Double check everything that the doctor said that the patient does not recall to ensure you have not missed anything.

References

1. Morley, G.D., *On functional elements of clause structure.* Word,, 1994. **45**(2): p. 119-124.

Morley, G. D. (1994). On functional elements of clause structure. *Word,, 45*(2), 119-124. doi:10.1080/00437956.1994.11435920
